# Dual Emission in the Near-Infrared and Visible Regions from a Mixed Cyanido-Bridged Eu^III^/Nd^III^(4-OHpy)-Co^III^ Layered Material

**DOI:** 10.1021/acs.inorgchem.2c01988

**Published:** 2022-09-26

**Authors:** Konstantinos Karachousos-Spiliotakopoulos, Vassilis Tangoulis, Anastasios Tasiopoulos, Nikos Panagiotou, Elefhteria Charalambous, Vassilis Nastopoulos, Sotirios Christodoulou

**Affiliations:** † Department of Chemistry, University of Patras, Patras 26504, Greece; ‡ Department of Chemistry, University of Cyprus, Nicosia 1678, Cyprus; § Inorganic Nanocrystals Laboratory, Department of Chemistry, University of Cyprus, Nicosia 1678, Cyprus; ∥ Experimental Condensed Matter Physics Laboratory, Department of Physics, University of Cyprus, Nicosia 1678, Cyprus

## Abstract

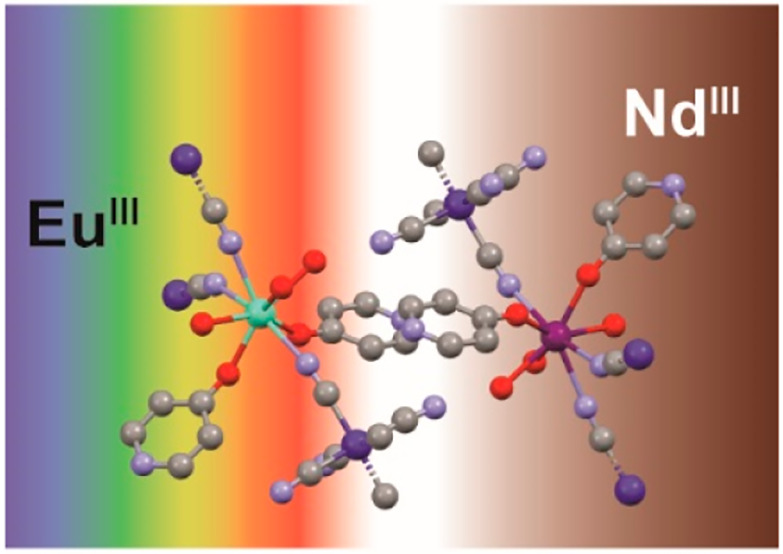

Coordination polymers (CPs) with a dual emission spanning from the visible (vis) to near-infrared (NIR) regions of the electromagnetic spectrum are used for optical sensors, medical diagnostics, and telecommunication technologies. We herein report the synthesis, structural characterization, and optical response of heterometallic cyanido-bridged layered {[Eu_*x*_Nd_*y*_(4-OHpy)_2_(H_2_O)_3_][Co(CN)_6_]} CPs, where 4-OHpy = 4-hydroxypyridine, with a multicolor emission profile across the vis and NIR regions. The crystals show an efficient energy transfer (ET) from the 4-OHpy ligand and the [Co(CN)_6_] ions to the Eu^3+^ and Nd^3+^ ions, resulting in an enhanced photoluminescence (PL) efficiency. We study the ET with steady-state and time-resolved PL, reporting an ET between the Ln^3+^ centers. The excitation-dependent emission of the mixed Ln^3+^ CPs and the control over the PL lifetime yield new insights into the optoelectronic properties of these materials.

## Abstract 2

A new series of Eu^3+^, Nd^3+^, and mixed Eu^3+^/Nd^3+^-layered coordination polymers were synthesized with strong emission in both the visible and near-infrared regions. The mixed Eu^3+^/Nd^3+^ shows an excitation-dependent emission profile and tunable radiative recombination rates.

Research on the development of materials with unique optical and magnetic properties^1−6^ has been the driving force for the development of an exciting new class of multifunctional supramolecular materials based on coordination polymers (CPs) for emerging optoelectronic devices. In fact, in the last decades, luminescence-based optical sensing has gained ground because it is a low-cost, nondestructible, highly versatile, and sensitive method. The materials for fluorescent sensing consist of emissive species, which are mainly transition- and/or lanthanide-metal ions.^7−10^ The sharp emission line widths from the f–f states of lanthanides, which span from the visible (vis) to near-infrared (NIR) electromagnetic spectrum, combined with the multiemissive transitions, which give access to multiple sensing wavelengths, have attracted the interest of the materials community.^11,12^ A new generation of luminescent materials incorporating the unique properties of lanthanide ions and the synthetic flexibility of CPs has emerged, paving the way for novel emerging technologies in the fields of medical theragnostics, imaging, and telecommunication.^11−17^ Especially for the case of materials with mixed lanthanide ions, dual and bimodal (UV/vis/NIR) emissive CPs,^18−24^ have been synthesized, accelerating the development of advanced technological applications in the areas of clinical diagnostics and ratiometric thermometers.^25−31^ The dual emission in CPs usually originates from mixed lanthanide ions, which provide the final dual emission bands. The distribution of the emissive centers in the CP is not trivial, and it has been the center of research over the years.^32^ Hence, the atactic distribution of lanthanide ions in the CP crystal structure directly influences its optical response and therefore its sensing efficiency.^33−35^

Recently, the role of the lanthanide ion, combined with pyridine derivatives and the red emissive linker [Co^III^(CN)_6_]^3–^, has been investigated.^36,37^ With regard to emissive multifunctional 2D materials, the cases of Dy^III^(4-OHpy)-Co^III^,^38^ Tb^III^(4-OHpy)-Co^III^^38b^ and the mixed lanthanide Tb^III^_0.5_Dy^III^_0.5_(4-OHpy)-Co^III^^38b^ have been shown. These compounds were shown to be multifunctional materials combining dual photoluminescence (PL) and single-molecule magnetism properties. For all cases, it was found that visible emission was switchable by selected wavelengths of UV excitation light.^39^ Therefore, we would like to step forward and focus our synthetic efforts on novel CP materials with broad-band emission covering both the vis and NIR spectral regions. Hence, we investigated the synthesis, physicochemical characterization, and optical properties of the mixed lanthanide Eu^III^Nd^III^(4-OHpy)-Co^III^ systems. The combination of Eu^3+^, which shows a pronounced emission in the low-energy part of the vis region, with a characteristic sharp NIR emission of Nd^3+^ at 1025 nm allowed us to decouple the radiative transitions of the two metal ions, giving rise to broad-band flexible sensing materials. Therefore, we synthesized five layered cyano-bridged CPs, where three contain two lanthanide ions (Eu^3+^ and Nd^3+^) based on the stoichiometric ratio of the reaction of {Eu_0.2_Nd_0.8_(4-OHpy)_2_(H_2_O)_3_][Co(CN)_6_]} (**1**), {Eu_0.5_Nd_0.5_(4-OHpy)_2_(H_2_O)_3_][Co(CN)_6_]} (**2**), and {Eu_0.8_Nd_0.2_(4-OHpy)_2_(H_2_O)_3_][Co(CN)_6_]} (**3**) and two CPs containing only one type of lanthanide ion, {Eu(4-OHpy)_2_(H_2_O)_3_] [Co(CN)_6_]} (**4**) and {Nd(4-OHpy)_2_(H_2_O)_3_][Co(CN)_6_]} (**5**).

The crystal structure of compound **4** was determined by single-crystal X-ray crystallography, while the purity and confirmation of the crystal phase of all of the synthesized compounds (**1**–**5**) were determined by CHN, Fourier transform infrared (FTIR), and powder X-ray diffraction (PXRD) analyses. Various structural plots of compound **4** are shown in Figures 1 and S1–S3. Selected interatomic distances and angles are listed in Table S2. Its asymmetric unit contains ^1^/_2_ {[EuCo(CN)_6_(4-OHpy)_2_(H_2_O)_3_]} because the Eu^3+^ cation, the H_2_O molecule O2, together with the Co^3+^ cation, and four of the cyanido groups of the hexacyanocobaltate(III) anion lie on a mirror plane. Each Eu^3+^ ion is bridged to three neighboring [Co(CN)_6_]^3–^ ions by three cyanido groups (Eu–N≡C–Co). The 8-coordination of the Eu^III^ center is completed by two 4-OHpy ligands and three H_2_O molecules; thus, its coordination sphere is {Eu^III^O5N3} and is shown in Figure 1a. The type of coordination polyhedron around the Eu^3+^ center was evaluated using *SHAPE*(39) software; the so-named continuous shape measures approach allows one to numerically estimate how far a real coordination sphere of a metal center deviates from an ideal polyhedron. Of the accessible 8-coordinate polyhedra for metal ions, the triangular dodecahedron is the most appropriate for the description of the eight donor atoms around the Eu^3+^ metal center (Table S3). The same conclusion is also reached by applying the angular criteria proposed by Kepert.^40^ The 6-coordination of the Co^3+^ center in the hexacyanocobaltate(III) anion comprises three bridging and three terminal cyanido groups, forming an octahedral {Co^III^C6} coordination sphere, with the trans C–Co^III^–C angles being in the range 175.7(4)–176.2(4)°.

**Figure 1:**
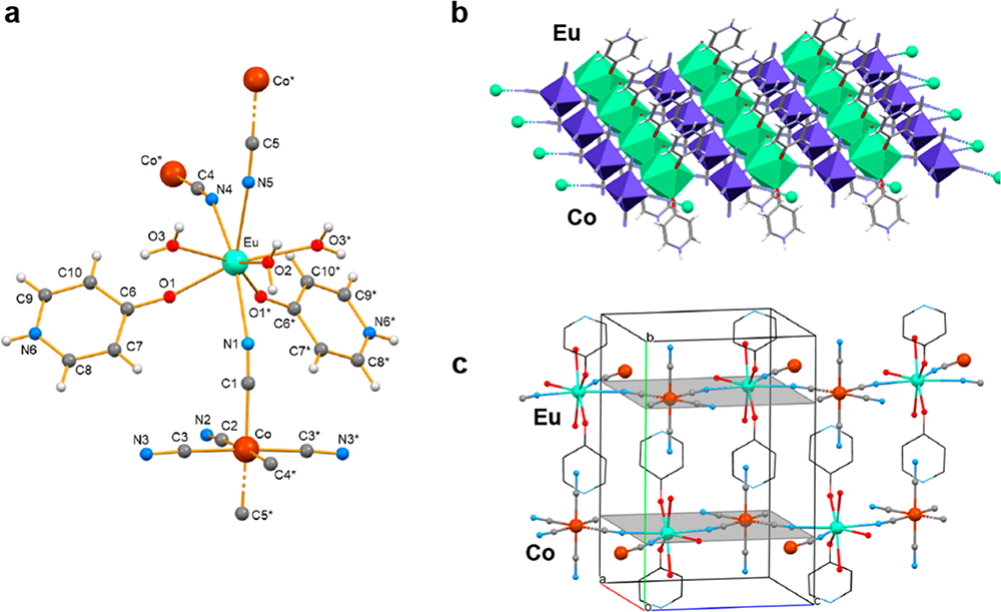
(a) Structural building unit {[Eu(4-OHpy)_2_(H_2_O)_3_][Co(CN)_6_]} of compound **4**. Atoms marked with an asterisk refer to symmetry-related atoms relative to those of the asymmetric unit. (b) Crystal structure of a single cyanido-bridged layer. (c) Layer arrangement in the polymeric crystal structure.

There is significant bending of the intermetallic cyanido bridges, as revealed by the Eu—N≡C angles of 158.5(8)° (Eu—N1≡C1), 150.8(9)° (Eu—N4≡C4), and 170.7(8)° (Eu—N5≡C5). This is likely due to the restrictions imposed on the Eu—N5≡C5—Co atoms to lie on the symmetry plane; further distortions are also necessary in order to allow for the proper coordination geometry on the Eu^3+^ and Co^3+^ metal centers (Figures 1 and S2 and S3). The cyanide-bridged polymeric structure is organized in layers that coincide with the mirror planes of the structure parallel to the *ac* plane and along the *b* axis (at *b* = 0.25 and 0.75). Pairs of symmetry-related ligands of 4-OHpy emerge from both sides of the layers, hampering polymerization in the third dimension along the *b* axis (Figure 1c). The coordinated H_2_O molecules, the terminal cyanido groups, and the pyridine NH groups of the ligands of 4-OHpy, which occur because of the presence of the dominant tautomer of the organic ligand,^38^ form strong intermolecular hydrogen bonds within each layer, as well as between adjacent layers, toward a robust 3D assembly (Table S4 and Figure 1c). No lattice solvent (crystallization) H_2_O molecules have been found.

The polycrystalline CP powders have been further probed structurally with PXRD. In Figure S1, the experimental XRD patterns of all of the pure and mixed lanthanide CPs are compared with the simulated patterns from the crystal structure of compound **4**. In addition, the IR spectra of the reported compounds have been recorded and are shown to exhibit the expected bands on the basis of the crystal structure of compound **4** (Figure S4).

The ratio of the mixed lanthanide CPs has been determined with microwave plasma atomic emission spectrometry (MP-AES), revealing that the ratio of the Eu^3+^ and Nd^3+^ precursor salts is translated quantitatively to the final CPs.

The optical properties of the polycrystalline samples have been studied thoroughly with both solid-state absorption and PL spectroscopy. The absorption profile of the solids has been probed with diffuse-reflectance spectroscopy (DRS), as shown in Figure 1a. The absorption spectra of all of the samples are dominated by strong absorption bands from both [Co(CN)_6_]^3–^ and 4-OHpy, which can be assigned to spin- and parity-forbidden electronic transitions.^41,42^ Hence, the absorption band at 330 nm originated from a singlet-to-singlet π → π* transition (^1^S_0_ → ^1^S_1_) and a d–d transition of Co^3+^ (^1^A_1g_ → ^1^T_1g_), whereas the higher-energy transition bands at 280 nm correspond to ^1^S_0_ → ^1^S_2_ and ^1^A_1g_ → ^1^T_2g_ from 4-OHpy and the Co^3+^ ion, respectively. In addition, the absorption tail down to 470 nm arises from spin-forbidden transitions of both 4-OHpy and Co^3+^.

The absorption peaks of the Nd^3+^ ion appearing in the spectra of compounds **2** and **5** correspond to the transitions from the ground state ^4^I_9/2_ to energetically higher states.^43^ In particular, the Nd^3+^ peaks were assigned as follows: ^4^I_9/2_ → ^4^D_3/2_ + ^4^D_5/2_ + ^4^D_1/2_ + ^2^I_11/2_ (355 nm), ^4^I_9/2_ → ^2^P_1/2_ (430 nm), ^4^I_9/2_ → ^2^G_9/2_ + ^2^D_3/2_ (460 nm), ^4^I_9/2_ → ^4^G_11/2_ + ^2^K_15/2_ (475 nm), ^4^I_9/2_ → ^4^G_7/2_ (512 nm), ^4^I_9/2_ → ^4^G_9/2_ + ^2^K_13/2_ (524 nm), ^4^I_9/2_ → ^4^G_5/2_ + ^2^G_7/2_ (582 nm), ^4^I_9/2_ → ^2^Η_11/2_ (631 nm), ^4^I_9/2_ → ^4^F_9/2_ (682 nm), ^4^I_9/2_ → ^4^S_3/2_ + ^4^F_7/2_ (745 nm), ^4^I_9/2_ → ^4^F_5/2_ + ^2^H_9/2_ (800 nm), ^4^I_9/2_ → ^4^F_3/2_ (870 nm). All of the DRS spectra have been normalized at 300 nm to decouple the lanthanide loading dependence of the solids. In that frame, we see that the Eu^3+^ absorption strength seems relatively low compared with that of Nd^3+^ in compound **2**, which is dominated by the Nd^3+^ transitions. Surprisingly, the absorption spectra of all of the CP compounds show a characteristic sharp peak at 1420 nm, which does not originate from either the Ln^3+^ ion or the 4-OHpy ligand and the [Co^III^(CN)_6_]^3–^ linker (Figure S5). Thereby, we assume that it is a state that arises from the coordinated ligand and/or linker. The emission of Eu^3+^ displays the characteristic PL peak at 614 nm, which corresponds to the ^5^D_0_ → ^7^F_2_ transition (Figure 2b). The PL spectrum from Nd^3+^ shows a main emission in the NIR region at 1025 nm related to the ^4^F_3/2_ → ^4^I_11/2_ radiative relaxation pathway, while the emissive recombinations at 891 and 1320 nm are assigned to ^4^F_3/2_ → ^4^I_9/2_ and ^4^F_3/2_ → ^4^I_13/2_, respectively.^44^ The weak emission from 4-OHpy and [Co(CN)_6_]^3–^ provides an indication of an effective energy transfer (ET) to the Ln^III^ metal ions.

**Figure 2:**
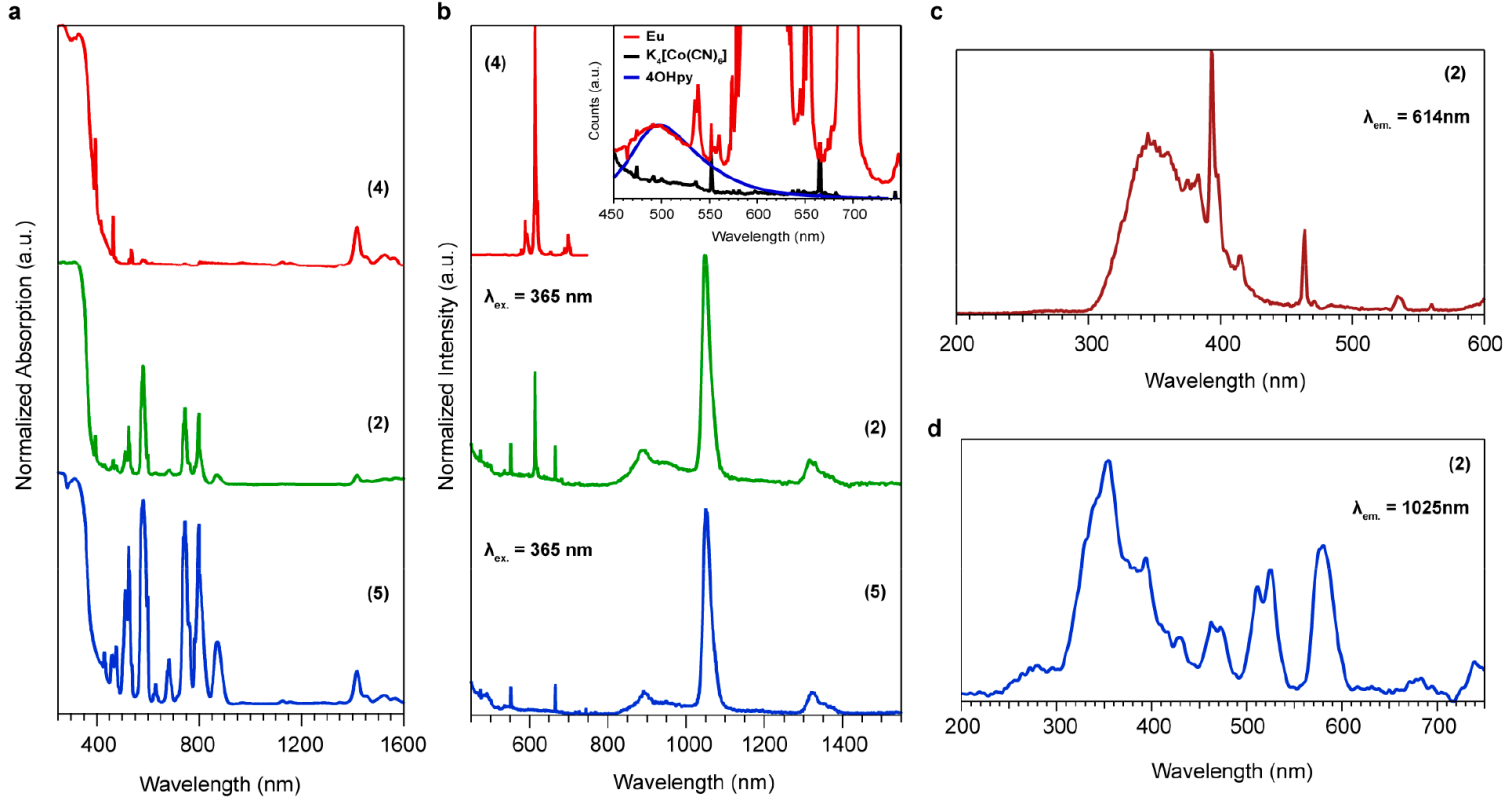
(a) Solid-state (diffuse-reflectance) electronic spectra of compounds **2**, **4**, and **5**. (b) PL spectra of compounds **2**, **4**, and **5** along with the emission of the ligands (inset). (c) PL excitation spectra of compound **2** with the emission centered at 614 nm probing the Eu^3+^ transition and (d) at 1024 nm for the Nd^3+^ transition.

The mixed lanthanide CPs are of particular interest both both Eu^3+^ and Nd^3+^ are optically active and the PL spectrum has strong emission in both the vis and NIR windows. The structure of the CPs implies that the Ln^III^ ions are well isolated in the structure and connected only with [Co(CN)_6_]^3–^ and 4-OHpy; thus, a direct Ln^III^-to-Ln^III^ ET should be forbidden. The first approach is to assume that the emission in CPs with only Eu^3+^ or Nd^3+^ is driven by ET from 4-OHpy and [Co(CN)_6_]^3–^ to the metal ion. In the case of mixed Ln^III^ CPs, the mechanism is rather more complicated because the photogenerated excitons from [Co(CN)_6_]^3–^ can be transferred to both Eu^3+^ or Nd^3+^ centers. Delving into the emission mechanism, we probed the excitation profile of the main transitions of both metals, ^5^D_0_ → ^7^F_2_ at 614 nm (Eu^III^) and ^4^F_3/2_ → ^4^I_11/2_ at 1025 nm (Nd^3+^) of compound **2**. The excitation spectra in Figure 2c show all of the electronic states that contribute to the emission of Eu^III^ at 614 nm and in Figure 2d for the Nd^3+^ at 1025 nm accordingly. In both excitation spectra, the broad absorption feature centered at 350 nm reflects the ET from 4-OHpy to Ln^III^. Interestingly, the high absorption cross sections of the ^7^F_0_ → ^5^L_6_ and ^7^F_0_ → ^5^D_2_ transitions of Eu^3+^ at 395 and 465 nm, respectively, point toward an efficient intraband relaxation in the metal, which leads to a strong emission at 614 nm. The same trends are followed in Nd^3+^, probing the excitation profile of the ^4^F_3/2_ → ^4^I_11/2_ at 1065 nm (Figure 2d). In the case of Nd^3+^ emission, the intraband relaxation appears to be the dominant mechanism, mainly because of the high oscillator strength of the ground state ^4^I_9/2_ absorption inside the 4f^3^ electronic configuration. The hypersensitive character of the ^4^G_5/2_ multiplet is translated to multiple peaks in the vis region at 512, 524, and 582 nm, in line with the absorption spectra (Figure 2a).^45^ It is worth noticing the absorption feature around 355 nm, which arises from the ^4^D_5/2_ multiplet; despite its strong character in the excitation spectra, the DRS measurements could not resolve it. The traditional relatively low absorption strength of the ^4^D_5/2_ multiplet in Nd^3+^ is in contrast with the excitation spectra, in which it seems to be the most efficient emission pathway. Hence, we assume that the intraband absorption adds constructively to ligand-to-metal ET for the NIR emission.

Interestingly, the excitation spectra of Nd^3+^ show a peak at 395 nm, which is not correlated with the electronic structure of the metal, pointing out that a hot exciton from another CP site contributes to the emission at 1024 nm. The energy gap of 3.14 eV matches with the ^7^F_0_ → ^5^L_6_ transition from Eu^3+^, suggesting that the photogenerated carriers from Eu^3+^ are efficiently transferred to the Nd^3+^ emissive sites. Elucidating the metal–bridge–metal ET, we collected the excitation profiles of the mixed Ln^III^ CPs shown in Figure 3a. We notice that when increasing the amount of Eu^3+^ in the material, the peak at 395 nm rises up linearly while the strength of the peak that is correlated with the ^4^D multiplet from Nd^3+^ fades. The inset in Figure 3a follows the peak intensity of these two transitions, indicating that the peak originating from the electronic states of Nd^3+^ decreases, lowering the amount of Nd^3+^ in the mixed Ln^3+^ CPs, and, on the other hand, the peak at 395 nm grows at the same rate as the Eu^3+^ content, so we can surmise that it is correlated with the ^7^F_0_ → ^5^L_6_ transition. In order to further support our model, time-resolved PL data have been collected in all of the CPs **1**–**5**, probing the ^5^D_0_ → ^7^F_2_ emission at 614 nm in Figure 3b. In line with the excitation spectra, the lifetime measurements suggest that the ET to Nd^3+^ ions originates from europium high energy states. The lifetime traces show a direct dependence of the Nd^3+^ metal centers on the Eu^3+^ emission. The recombination rate of the ^5^D_0_ → ^7^F_2_ transition decreases, while the CPs are loaded with Nd^3+^, further supporting the ET mechanism with exciton lifetimes spanning from 1.4 μs for pure Eu^3+^-based CPs to 41 μs for compound **1**. Moreover, the dual emission profile of the mixed Ln^3+^ CPs is excitation-sensitive; thus, we describe in Figure 3c the relative percentages of the emission in both the IR and vis regions correlated with the excitation energy. In Figure 3c, we show the normalized excitation sensitivity of compound **2**, indicating that in certain excitation energies we can selectively excite the Nd and/or Eu emissive center, gaining control over the relative dual emission of the CPs.

**Figure 3:**
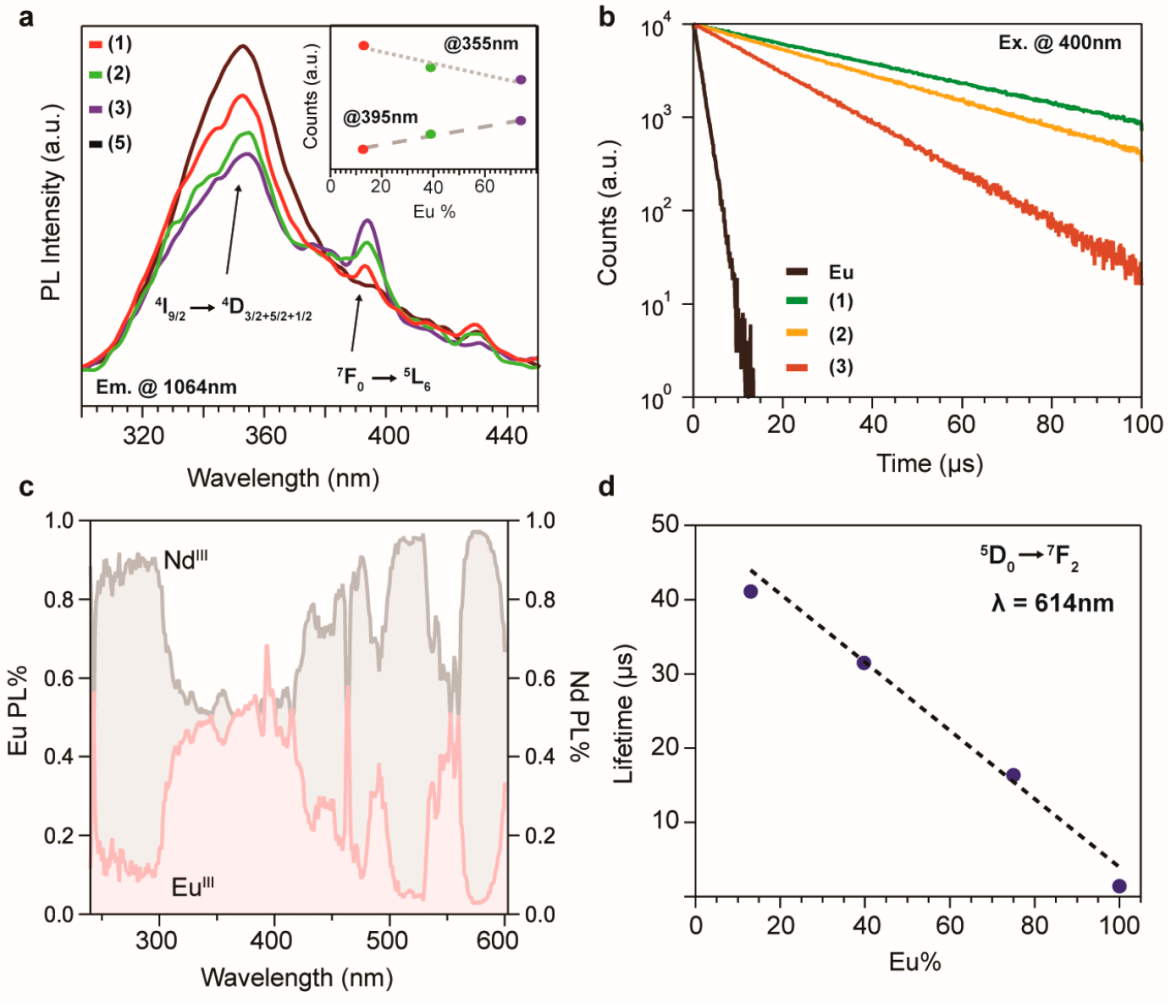
(a) Excitation spectra of the mixed Ln^3+^ CPs. (b) Lifetime traces of the Eu^3+^ emission at 614 nm excited with a picosecond laser at 400 nm. (c) Excitation-dependent PL intensity of the mixed Ln^3+^ CPs. (d) Summarized lifetime values in terms of the europium content.

In summary, we report a new series of layered cyanido-bridged CPs, with heterometallic emissive centers and dual photoluminescence in both the IR and vis spectral regions. Using excitation spectroscopy and time-resolved spectroscopy, we elucidated the photochemical mechanism, demonstrating tunable emission rates by adjusting the Nd^3+^ percentage in the crystals. The wide spectral coverage, together with control of the emission profile of the CPs, paves the way for the next generation of materials that can be deployed in optical sensing and imaging devices.

## Supporting Information Available

Detailed experimental procedures, crystallographic data, PXRD patterns, MP-AES analysis, and FTIR spectra for all of the samples (PDF)
