# Supplementary material for: Dual Emission in the Near-Infrared and Visible Regions from a Mixed Cyanido-Bridged EuIII/NdIII(4-OHpy)-CoIII Layered Material
Source: Inorg Chem. 2022 Sep 26;61(40):15806–11. doi: 10.1021/acs.inorgchem.2c01988 (PMC9554905; doi:10.1021/acs.inorgchem.2c01988)
Supplement: Supplementary file 1 — ic2c01988_si_001.pdf [file ic2c01988_si_001.pdf]

Supporting Information

Dual emission in the near-infrared and visible from mixed cyanido bridged Eu^{III}/Nd^{III} (4-hydroxypyridine)-Co^{III} layered material

Konstantinos Karachousos-Spiliotakopoulos[1], Vassilis Tangoulis[1], Anastasios Tasiopoulos[2], Nikos Panagiotou[2] ,Elefhteria Charalambous[3,4], Vassilis Nastopoulos[1], Sotirios Christodoulou[3]**

1. Department of Chemistry, University of Patras, 26504 Patras, Greece
2. Department of Chemistry, University of Cyprus, 1678 Nicosia, Cyprus
3. Inorganic Nanocrystals Laboratory, Department of Chemistry, University of Cyprus, 1678 Nicosia, Cyprus
4. Experimental Condensed Matter Physics Laboratory, Department of Physics, University of Cyprus, Nicosia 1678, Cyprus

Email: christodoulou.sotirios@ucy.ac.cy
vtango@upatras.gr

EXPERIMENTAL SECTION

Materials, Physical Techniques and Spectroscopic Methods. All manipulations were performed under aerobic conditions. $DyCl_3 \cdot 6H_2O$, $K_3[Co(CN)_6]$ and 4-OHpy were purchased from commercial sources. Elemental analyses were performed by the University of Patras microanalytical service. FT-IR spectra (4000-450 cm^{-1}) were recorded using a Perkin-Elmer 16PC spectrometer with samples prepared as KBr pellets.

Powder XRD measurements.The powder X-ray diffraction (P-XRD) measurements were performed at room temperature on a Malvern Panalytical X'Pert PRO diffractometer with focusing Kα1 geometry. Polycrystalline samples were loaded in 1 mm borosilicate glass capillaries while the X-ray tube operated at 45 kV and 40 mA. The incident-beam side (CuKα1 radiation, λ = 1.54056 Å) is equipped with a focusing X-ray mirror, a 0.5° fixed divergence slit, 0.5° anti-scatter slits and 0.04 rad Soller slits, while on the diffracted-beam side the system was configured with 0.04 rad Soller slits

and a PIXcel1D detector with anti-scatter shielding. Four scans were performed in Debye-Scherrer mode, with a step size of 0.0066 ˚on a spinning stage (~300 rpm), within a 2θ range of 4.0–90.0˚. No radiation damage was observed even after 5 h of measurement, therefore all scans were merged together to increase counting statistics.

MPA-AES

10 mg of sample was weighted in a 16 mm glass digestion tube and 4 mL of a mixture of concentrated nitric acid : hydrochloric acid (1:1) were added. The mixture was digested at 150 ºC in thermoreactor CR3200 (WTW Xylem Analytics, Germany) until sample fully dissolved. The solution was diluted to a 20 mL volumetric flask with Millipore water. The final solution were further diluted 10 times prior to measurement. The analysis was performed using Microwave Plasma Atomic Emission Spectrometry (MP-AES 4210, Agilent Technologies) equipped with a Oneneb2 nebulizer and a double-pass glass cyclonic spray chamber. The conditions of measurement as follows: uptake time 15s, stabilization time 15s, read time 3s, number of replicates 3, nebulizer flow: 0.75 L/min, wavelegths: Eu(381.967 nm), Nd (430.358 nm). For quantification purposes, a 6-point calibration curve was constructed using standard solutions corresponding to concentration of 0.1, 0.5, 1.0, 2.5, 5.0, 10 mg/L.

Optical spectroscopy.

Diffuse reflectance spectra were obtained with an Agilent Cary 5000 instrument with the use of an integrating sphere. A KBr reference pellet was prepared by grinding pure KBr and the polycrystalline samples of the sample in a pestle and mortar until a homogenous fine powder was obtained. Solid-state, room-temperature photoluminescence emission and excitation spectra were performed using a Horiba Jobin Yvon iHR550 Fluorolog system coupled with a Hamamatsu RS5509-73liquid-nitrogen cooled photomultiplier. All the samples were encapsulated in a soda lime glass substrate and excited at 405 nm (70 mW) with a continuous-wave (CW) laser for the collection of the PL spectra. The excitation spectra were excited with the Xenon lamped coupled with a monochromator while for time-resolved measurements a pulsed laser diode was employed (Horiba Nanoled, λ = 405 nm, pulse full-width-half-maximum of 50 ps, fluence ≈ 1 nJ/cm2).

Preparation of [$EuCo(CN)_6(4\text{-}OHpy)_2(H_2O)_3]_n$ (4). To a warm, pale yellow solution of $Eu(NO_3)_3{\cdot}6H_2O$ (0.053 g, 0.12 mmol) in distilled H_2O (0.5 mL) was added solid white 4-OHpy (23.0 mg, 0.24 mmol) to get solution **1**. Subsequently, $K_3[Co(CN)_6]$ (40.0 mg, 0.12 mmol) was dissolved in distilled H_2O (0.5 mL) in order to obtain solution **2**. Then, the solution **2** was quickly added to the solution **1** under stirring. The resulting pale yellow solution was stored in a closed vial at room temperature. After one day needle-like shaped crystals appeared. The crystalline product was collected via filtration. Analytical data calculated for $C_{16}H_{16}N_8O_5Eu$ (found values are in parentheses): C 34.79 (34.98), H 2.92(2.86), N 20.29(20.17) %. IR bands (KBr, cm^{-1}): 3610s, 3529w, 3314sh, 3264s, 3153s, 3085w, 2972w, 2644w, 2179m, 2166m, 2154s, 2134s,1637s, 1590m, 1541s, 1516s, 1386s, 1284w, 1246w, 1188s, 1078w, 1033w, 999s, 972w, 872m, 858m, 773s, 661w, 604m, 562s, 503m, 451m, 436m.

Preparation of [$NdCo(CN)_6(4\text{-}OHpy)_2(H_2O)_3]_n$ (5). The compound **(5)** was synthesized by replacing $Eu(NO_3)_3{\cdot}6H_2O$ with $Nd(NO_3)_3{\cdot}6H_2O$ (0.053 g, 0.12 mmol) with the same experimental procedure as compound (**4**). Crystals of very poor quality have been obtained. Analytical data calculated for $C_{16}H_{16}N_8O_5Nd$ (found values are in parentheses): C 35.29 (34.47), H 2.96(2.87), N 20.58(20.36) %.

Preparation of [$Eu_xNd_{1-x}Co(CN)_6(4\text{-}OHpy)_2(H_2O)_3]_n$. The Eu/Nd-doped complexes were synthesized with the same experimental procedure as compound **(4)**, and the total amount of Eu^{3+} and Nd^{3+} ions was 0.12 mmol. The employed experimental molar ratios of Eu^{3+} to Nd^{3+} were 80:20, 50:50, 20:80, corresponding to the **3**, **4** and **5** respectively for the Eu/Nd-doped complexes. The molar ratios of Eu^{3+} to Nd^{3+} of the products were calculated through EDS.

[$Eu_{0.74}Nd_{0.26}Co(CN)_6(4\text{-}OHpy)_2(H_2O)_3]_n$ (**3**). Analytical data calculated for $C_{16}H_{16}N_8O_5Eu_{0.74}Nd_{0.26}$ (found values are in parentheses): C 34.92 (35.07), H 2.93(2.82), N 20.36(20.28) %.

[$Eu_{0.39}Nd_{0.61}Co(CN)_6(4\text{-}OHpy)_2(H_2O)_3]_n$ (**2**). Analytical data calculated for $C_{16}H_{16}N_8O_5Eu_{0.39}Nd_{0.61}$ (found values are in parentheses): C 35.09 (35.21), H 2.95(2.87), N 20.46(20.29) %.

[$Eu_{0.13}Nd_{0.87}Co(CN)_6(4\text{-}OHpy)_2(H_2O)_3]_n$ (**1**). Analytical data calculated for $C_{16}H_{16}N_8O_5Eu_{0.13}Nd_{0.87}$ (found values are in parentheses): C 35.22(35.31), H 2.96(2.85), N 20.54(20.46) %.

Single-Crystal X-ray Crystallography. A single crystal of compound **1** coated with paratone-N oil was scooped up in cryo-loops at the end of a copper pin. Diffraction data were collected by

the *ω*-scan technique on a SuperNova X-ray diffraction system from Rigaku under a stream of nitrogen gas at 100(2) K using Cu*Kα* (λ = 1.5418 Å) radiation. Data were collected and processed by the *CRYSALIS CCD* and *RED* software,[1] respectively. The reflection intensities were corrected for absorption by the multi-scan method. The structure was solved using direct methods with *SIR92*[2] and *SHELXT-2018/2*[3] and refined by full-matrix least-squares on F^2 with *SHELXL-2018/3*.[4] The crystal structure was refined as a non-merohedral twin with a domain ratio of 72:28. All non-H atoms were refined anisotropically. Appropriate restraints/constraints have been applied during refinement on the anisotropic displacement parameters of some atoms to handle the symptoms of the twinning; the presence of some difference Fourier map peaks are also known characteristic symptoms of non-merohedrally twinned crystals. Carbon-bound H-atoms were included in calculated positions (riding model). All H-atoms of the coordinated solvent water molecules O2 and O3 together with that on the N6-atom of the 4-hydroxypyridine ligand were located in difference Fourier maps and refined isotropically applying soft distance restraints (DFIX) to ensure the proper geometry. Geometric/crystallographic calculations were carried out using *WINGX*[5], *PLATON*[6] and *OLEX2*[7] packages; molecular/packing graphics were prepared with *MERCURY*.[8] Experimental details are listed in Table S1. The structural data have been deposited at the CCDC database (reference number 2177057).

REFERENCES

1 *CrysAlisPRO*, 1.171.41.93a; Rigaku Oxford Diffraction, 2020.

2 Altomare, A.; Cascarano, G.; Giacovazzo, C.; Guagliardi, A.; Burla, M. C.; Polidori G.; Camalli, M. *SIR92* – a program for automatic solution of crystal structures by direct methods. *J. Appl. Crystallogr.* **1994**, *27*, 435.

3 Sheldrick, G. M. *SHELXT* – integrated space-group and crystal-structure determination. *Acta Crystallogr*. **2015**, *A*71, 3–8.

4 Sheldrick, G. M. Crystal structure refinement with *SHELXL*. *Acta Crystallogr*. **2015**, C*71*, 3–8.

5 Farrugia, L. J. *WinGX* and *ORTEP for Windows*: an update. *J. Appl. Crystallogr*. **2012**, *45*, 849–854.

6 Spek, A. L. Structure validation in chemical crystallography. *Acta Crystallogr*. **2009**, D*65*, 148–155.

7 Dolomanov, O. V.; Bourhis, L. J.; Gildea, R. J.; Howard, J. A. K.; Puschmann, H. *OLEX2*: a complete structure solution, refinement and analysis program. *J. Appl. Crystallogr*. **2009**, *42*, 339–341.

8 Macrae, C. F.; Sovago, I.; Cottrell, S. J.; Galek, P. T. A.; McCabe, P.; Pidcock, E.; Platings, M.; Shields, G. P.; Stevens, J. S.; Towler, M.; Wood, P. A. *Mercury 4.0*: from visualization to analysis, design and prediction. *J. Appl. Crystallogr.* **2020**, *53*, 226–235.

Table S1. Crystal data and structure refinement for compound **1**.

Chemical formula	$C_{16}H_{16}CoEuN_8O_5$
Formula weight	611.26
Temperature [K]	100(2)
Crystal system	Monoclinic
Space group	$P2_1/m$
a [Å]	6.9255 (4)
b [Å]	15.7271 (9)
c [Å]	9.6583 (5)
β [deg]]	101.891 (5)
Volume [Å^3]	1029.39 (10)
Z	2
ρ_{calc} [g cm^{-3}]	1.972
F(000)	596
Radiation type	Cu $K\alpha$
μ [mm^{-1}]	28.32
Collected reflections	1907
Unique reflections	1907
θ/completeness (%)	67.0/99.8
Data/parameters/restraints	1907/167/39
Final R_1, $wR_2(F^2)$ [$I > 2\sigma(I)$]	0.053, 0.140
Final R_1, $wR_2(F^2)$ (all data)	0.064, 0.150
Goodness-of-fit on F^2	1.053
$\Delta\rho_{max}$, $\Delta\rho_{min}$ (e Å^{-3})	4.470, −1.772
CCDC number	2177057

Table S2. Selected bond lengths [Å] and angles [°] for compound **4**.

Bond lengths			
Eu—O1	2.308 (5)	Co—C1	1.906 (10)
Eu—O2	2.520 (7)	Co—C2	1.878 (10)
Eu—O3	2.409 (6)	Co—C3	1.894 (9)
Eu—N1	2.527 (9)	Co—C4	1.917 (10)
Eu—N4	2.539 (9)	Co—C5^{ii}	1.869 (11)
Eu—N5	2.523 (9)		
Bond angles			
O1—Eu—O1^{i}	75.6 (3)	C1—Co—C4^{iii}	97.1 (4)
O1—Eu—O3^{i}	73.9 (2)	C2—Co—C1	86.9 (4)
O1^{i}—Eu—O3^{i}	149.5 (2)	C2—Co—C3	88.9 (2)
O1^{i}—Eu—O3	73.9 (2)	C2—Co—C4^{iii}	176.0 (4)
O3^{i}—Eu—O3	136.6 (3)	C3—Co—C1	91.5 (2)
O1—Eu—O2	131.64 (17)	C3^{i}—Co—C3	176.2 (4)
O3—Eu—O2	71.98 (17)	C3—Co—C4^{iii}	91.0 (2)
O1—Eu—N5	131.35 (19)	C5^{ii}—Co—C1	175.7 (4)
O3—Eu—N5	71.89 (17)	C5^{ii}—Co—C2	88.8 (4)
O2—Eu—N5	66.0 (3)	C5^{ii}—Co—C3	88.4 (2)
O1—Eu—N1	81.9 (2)	C5^{ii}—Co—C4^{iii}	87.2 (4)
O3—Eu—N1	94.0 (2)	N1—C1—Co	173.8 (9)
O2—Eu—N1	67.5 (3)	N2—C2—Co	178.6 (9)
N5—Eu—N1	133.5 (3)	N3—C3—Co	176.0 (6)
O1—Eu—N4	80.7 (2)	N4—C4—Co^{iv}	176.1 (11)
O3—Eu—N4	94.2 (2)	N5—C5—Co^{v}	174.8 (9)
O2—Eu—N4	134.6 (3)	C1—N1—Eu	158.5 (8)
N5—Eu—N4	68.5 (3)	C4—N4—Eu	150.8 (9)
N1—Eu—N4	157.9 (3)	C5—N5—Eu	170.8 (8)

Symmetry codes: (i) x, $-y$+3/2, z; (ii) x−1, y, z−1; (iii) x, y, z−1; (iv) x, y, z+1; (v) x+1, y, z+1.

Table S3. Coordination geometry analysis for the Eu metal centre of compound **4** by the SHAPE v2.1 software.

```
8-coordinated Dy complex

CShM value  Symmetry   Polyhedron
-----------------------------------------------------------------------------------
  32.62      D8h       Octagon
  24.77      C7v       Heptagonal pyramid
  17.05      D6h       Hexagonal bipyramid
  11.04      Oh         Cube
   3.25      D4d       Square antiprism
   0.54      D2d       Triangular dodecahedron
  13.73      D2d       Johnson gyrobifastigium J26
  30.56      D3h       Johnson elongated triangular bipyramid J14
   3.59      C2v       Biaugmented trigonal prism J50
   2.81      C2v       Biaugmented trigonal prism
   3.09      D2d       Snub diphenoid J84
  11.70      Td         Triakis tetrahedron
  26.27      D3h       Elongated trigonal bipyramid
```

Table S4 Geometry (Å, °) of the strong hydrogen-bonding motifs in compound **4**.

D–H···*A*	*D*–H	H···*A*	*D*···*A*	*D*–H···*A*
O2–H2A···N2^{i}	0.85(2)	1.97(4)	2.782(11)	160(9)
O2–H2B···O1^{i}	0.85(4)	2.49(3)	3.221(8)	145(2)
O3–H3A···N3^{ii}	0.85(5)	1.84(5)	2.677(10)	168(9)
N6–H6···N2^{iii}	0.87(3)	2.17(4)	3.023(9)	167(6)

Symmetry codes: (i) *x*+1, *y*, *z*; (ii) –*x*+1, *y*+1/2, –*z*+1; (iii) –*x*, *y*–1/2, –*z*+1.

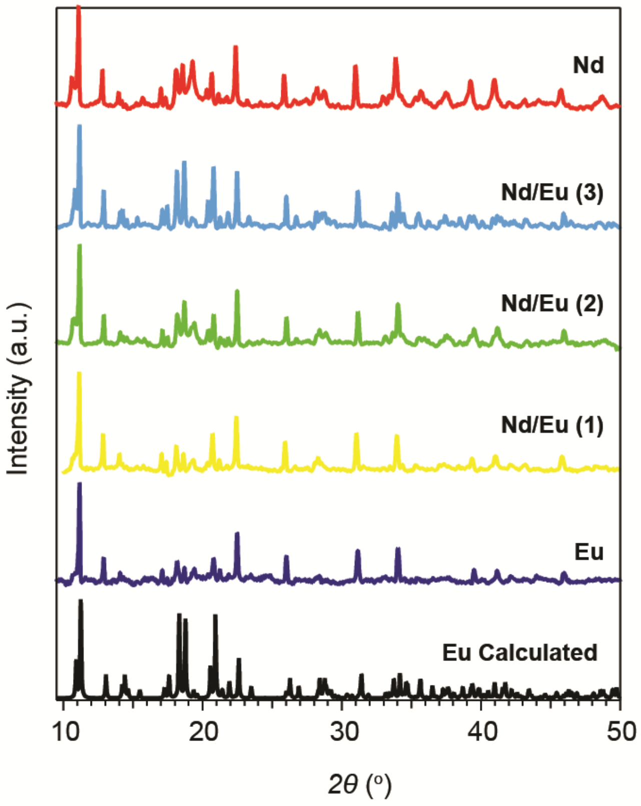

Figure S1. Experimental and theoretical X-ray diffraction patterns of both mixed and mono-metallic coordination polymers.

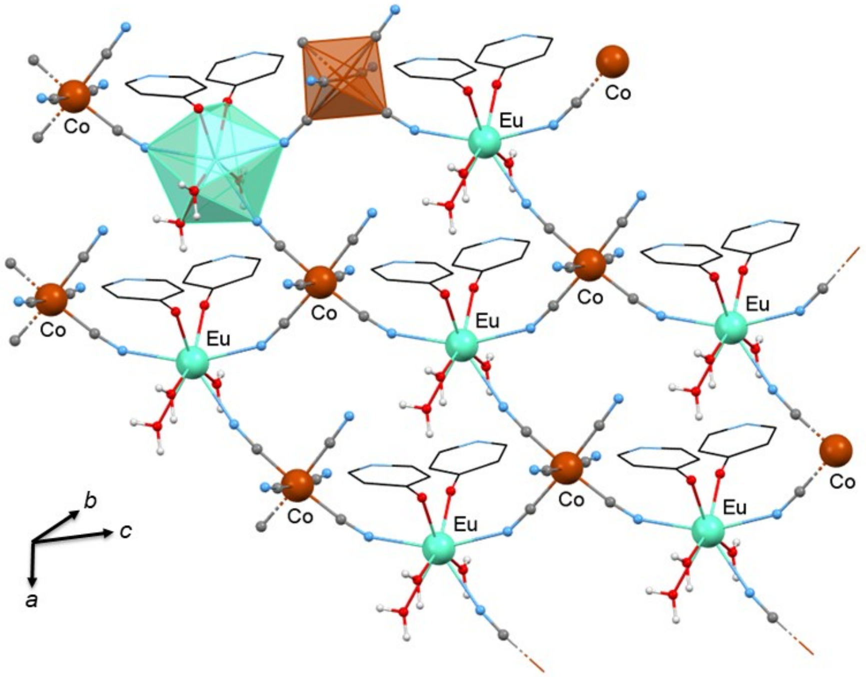

Figure S2. A layer of the cyanide-bridged polymeric structure of compound 4 developed parallel to the *ac* plane. Hydrogen atoms are only shown for the coordinated water molecules.

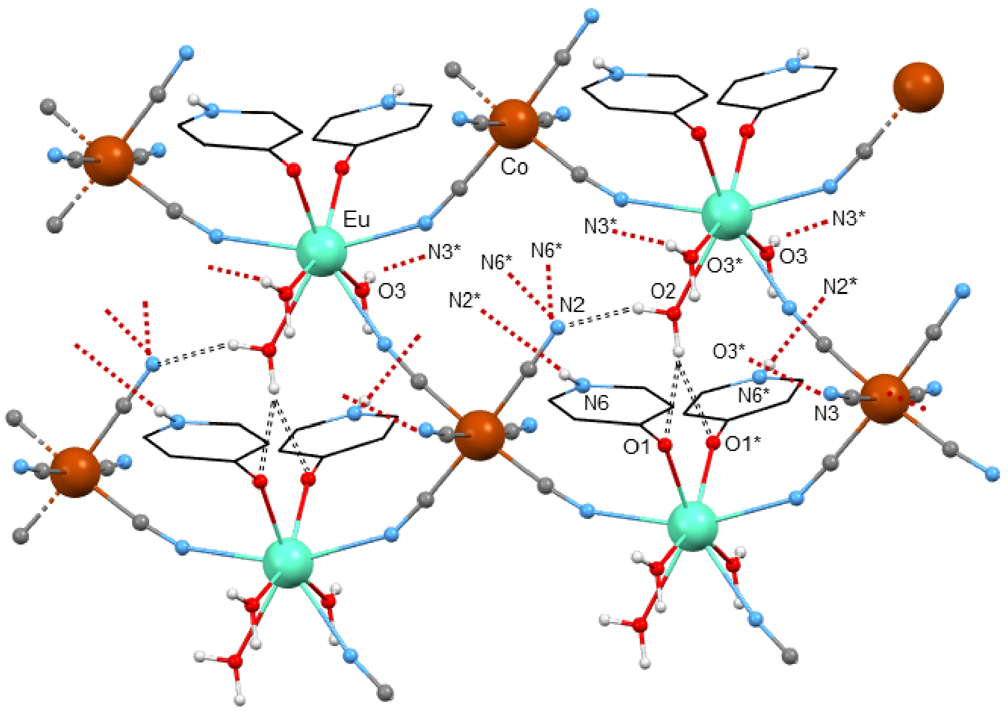

Figure S3. Part of a layer of the cyanide-bridged polymeric structure of compound **4**. The intermolecular interactions connecting the molecules on the layer are shown in double black dashed lines; hanging contacts to atoms on the adjacent layers are shown in red dashed lines. Atoms marked with an asterisk refer to symmetry-related atoms. Only contact hydrogen atoms are drawn.

FT-IR

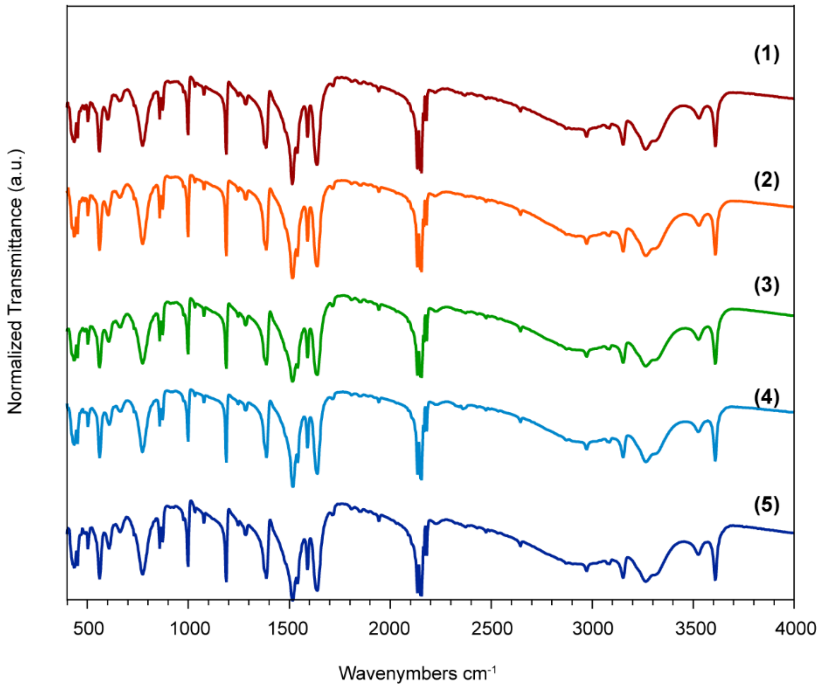

Figure S4. The stretching vibrations 2179m, 2166m, 2154s, 2134s can be attributed to the presence of CN^- moieties in both bridging and terminal modes. The band 1637s can be assigned to $\delta(H_2O)$. The bands 1590m, 1541s, 1386s, 999s, can be assigned to C=C, C=N stretching modes of the pyridine ring.The band 1516s can be assigned to the stretching mode of C=O which occurs due to isomerization of 4-hydroxy-pyridine to 4-pyridinone. This band does not appear in the IR spectra of the uncoordinated organic ligand. 1284w, 1246w, 1188s, 872m, 858m C-H and ring deformation.

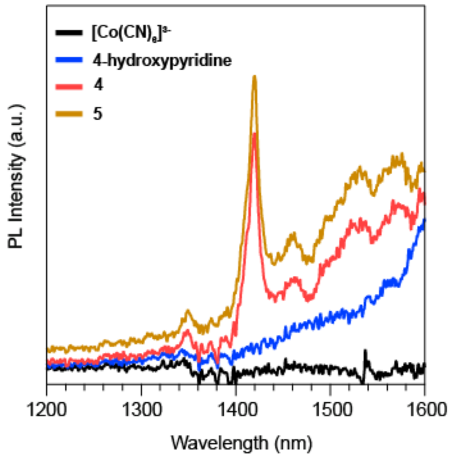

Figure S5 Absorption spectra of the CP compounds 4 and 5, the ligand and the linker.

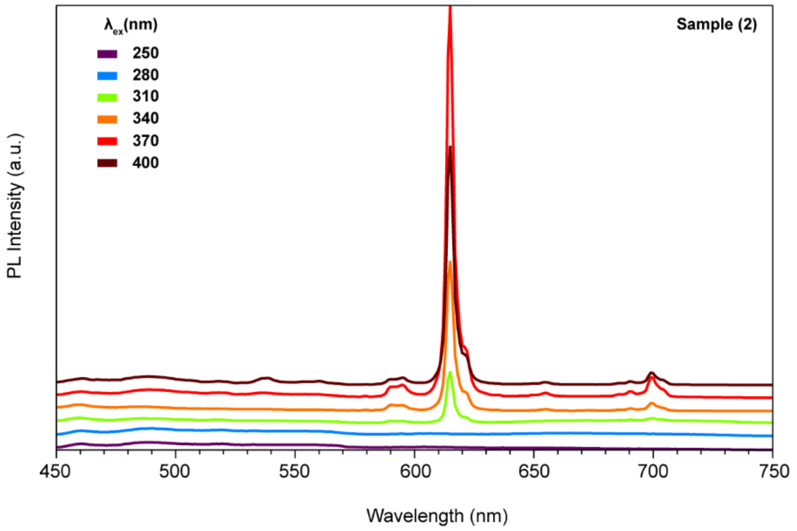

Figure S6 Photoluminescence spectra of compound 2 under various excitation wavelengths.
